# SARS-CoV-2 vaccine-associated subacute thyroiditis: insights from a systematic review

**DOI:** 10.1007/s40618-022-01747-0

**Published:** 2022-01-29

**Authors:** S. Ippolito, D. Gallo, A. Rossini, B. Patera, N. Lanzo, G. F. M. Fazzino, E. Piantanida, M. L. Tanda

**Affiliations:** 1 Endocrine Unit, ASST dei Sette Laghi, Viale Borri, 57, 21100 Varese, Italy; 2 Endocrine and Diabetes Unit, ASST Papa Giovanni XXIII, Bergamo, Italy; 3 Department of Medicine and Surgery, University of Insubria, Varese, Italy

**Keywords:** SARS-CoV-2, Vaccine, Thyroid, Subacute thyroiditis, Adjuvants

## Abstract

**Purpose:**

To perform a systematic review on published cases of subacute thyroiditis (SAT) secondary to SARS-CoV-2 vaccination, to highlight main features and increase the awareness of this condition.

**Methods:**

Original reports of SAT developed after SARS-CoV-2 vaccination (mRNA, viral vector, or inactivated virus vaccines) were retrieved from a search of electronic databases. Individual patient data on demographics, medical history, type of vaccine, workup and therapies were collected. Wilcoxon rank-sum, Kruskal–Wallis and chi-squared tests were employed for comparisons.

**Results:**

30 articles including 48 reports were retrieved, 3 additional cases evaluated by the Authors were described and included for analysis. Of the 51 patients, 38 (74.5%) were women, median age was 39.5 years (IQR 34–47). Patients developed SAT after a median of 10 days (IQR 4–14) after the vaccine shot. Baseline thyroid exams revealed thyrotoxicosis in 88.2% of patients, decreasing at 31.6% at follow-up. Corticosteroids were used in 56.4% of treated patients. Patients undergoing non-mRNA vaccines were most frequently Asian (*p* = 0.019) and reported more frequently weight loss (*p* = 0.021). All patients with a previous diagnosis of thyroid disease belonged to the mRNA vaccine group.

**Conclusion:**

SARS-CoV-2 vaccine-associated SAT is a novel entity that should be acknowledged by physicians. Previous history of thyroid disease may predispose to develop SAT after mRNA vaccines, but further studies and larger cohorts are needed to verify this suggestion. SARS-CoV-2 vaccine-associated SAT is usually of mild/moderate severity and could be easily treated in most cases, thus it should not raise any concern regarding the need to be vaccinated.

## Introduction

Subacute thyroiditis (SAT) is an inflammatory disease of the thyroid gland causing transient thyrotoxicosis, characterized by neck pain and symptoms of thyroid hormones excess. Despite disease presentation and clinical course are distinctive, etiology is not entirely determined.

Viral infections are considered the main trigger of SAT, especially for the frequent association with recent upper respiratory tract infections [1]. Main viruses associated with SAT are coxsackievirus A and B, echovirus, mumps, measles, influenza. Shortly after the onset of SARS-CoV-2 pandemic, many cases of SAT induced by this virus have been described [2–6]. Direct evidence of viral presence in the thyroid gland has been indeed demonstrated for several viruses [7], including SARS-CoV-2 [8, 9]. Genetic susceptibility seems to also have a key role in pathogenesis, since SAT is often associated with some HLA haplotypes, in particular HLA-B*35 [10].

SAT has also been described after vaccination against H1N1 vaccine [11], seasonal influenza virus vaccine [12, 13], Human Papillomavirus vaccine [14, 15], and hepatitis B vaccination [16]; more recently, a rising number of cases of SAT following SARS-CoV-2 vaccination has also been reported, whether for the nucleic acid (mRNA), the viral vector (adenovirus), or the inactivated virus vaccine. So far, some possible pathophysiological explanations for post-vaccination SAT have been proposed, yet distinct mechanisms remain unclear. The first mechanism suggested is the autoimmune/inflammatory syndrome induced by adjuvants (ASIA) [17], an adjuvants-triggered immune reaction due to vaccine (or other drugs or products) excipients which could determine dysregulation of both innate and adaptive immune systems, possibly causing destructive thyroiditis or even new onset of autoimmune diseases such as Hashimoto’s thyroiditis [18]. SAT as part of ASIA syndrome has been formerly described also for hepatitis B, Human Papillomavirus, and influenza vaccinations [19]. A second hypothesis is linked to molecular mimicry: a recent in-vitro study [20] found that antibodies against SARS-CoV-2 proteins could cross-react with several tissue antigens, including thyroid antigens such as thyroid peroxidase (TPO). This immune crosstalk could then represent a common mechanism for immune-mediated complications both after SARS-CoV-2 infections [21] and for SARS-CoV-2 vaccination.

Aim of this study is to perform a systematic review of published cases of SAT secondary to SARS-CoV-2 vaccination, to highlight the characteristics of this condition, increasing the awareness of this possible side effect, and identify if new mRNA vaccines may trigger this side effect with a different pattern.

## Methods

**Fig. 1:**
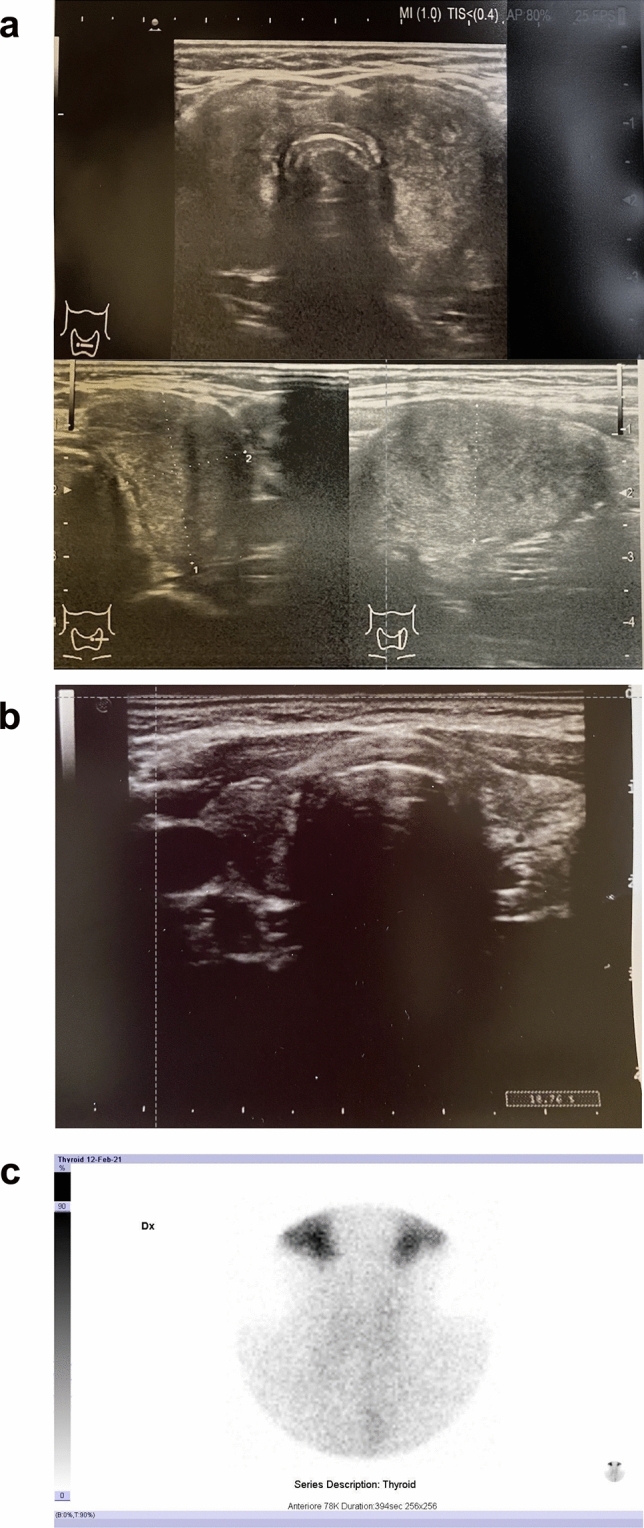
Thyroid ultrasound and scintigraphy in SARS-CoV-2 post-vaccination SAT. Ultrasound performed **a** at diagnosis and **b** at resolution of symptoms in a patient with subacute thyroiditis developed after mRNA vaccine for SARS-CoV-2 (Case 1, described in Table 1). At diagnosis thyroid was enlarged, highly inhomogeneous and nodular. After therapy, at resolution of symptoms, thyroid echostructure remained inhomogeneous, but thyroid volume was back to normal and there were no nodules. **c** Thyroid scintigraphy revealed low/absent thyroid uptake in a patient with subacute thyroiditis developed after mRNA vaccine for SARS-CoV-2 (Case 1, described in Table 1)

We performed a patient-level systematic review of case report and case series of SAT associated to SARS-CoV-2 vaccination reported in literature. Two additional unpublished cases evaluated in the Endocrine Unit of ASST dei Sette Laghi (Varese-Italy), and one case evaluated in the Endocrine and Diabetes Unit of ASST Papa Giovanni XXIII (Bergamo-Italy) are reported in Table 1 and Fig. 1 and were included in the database.

**Table 1:** Three cases of SAT occurred after SARS-CoV2 vaccination, Case 1 and 2 evaluated in Endocrine Unit, ASST dei Sette Laghi, Varese (Italy)—Case 3 evaluated in Endocrine and Diabetes Unit, ASST Papa Giovanni XXIII, Bergamo (Italy)

Case n. 1 (Varese)	Case n. 2 (Varese)	Case n. 3 (Bergamo)
**Basal features**		
Caucasian Italian female, 41 years. No history of thyroid disease	Caucasian Italian female, 31 years. History of seronegative hypothyroidism, well controlled with levothyroxine 50 mcg daily. Normal thyroid function in May 2021	Caucasian Italian female, 64 years No personal history of thyroid disease, a sister affected by hypothyroidism
Pfizer/BioNTech 1st dose 10th January 2021	Astra Zeneca 1st dose 15th March 2021	Pfizer/BioNTech 1st dose 28th April 2021
Pfizer/BioNTech 2nd dose 1st February 2021	Pfizer/BioNTech 2nd dose 28th June 2021	Pfizer/BioNTech 2nd dose 20th May 2021
Post-vaccination general symptoms: Mild general symptoms after 1st dose (fatigue, arm pain)	Post-vaccination general symptoms: Moderate general symptoms after 2nd dose (high fever, intense fatigue, headache)	Post-vaccination general symptoms: Fever after 2nd dose
Post-vaccination thyroid symptoms: Neck pain radiating to ear and jaw, fast heartbeat, sweating. Onset 15 days after 1st dose	Post-vaccination thyroid symptoms: Severe neck pain radiating to ear, fast heartbeat, sweating, headache. Onset 28 days after 2nd dose	Post-vaccination thyroid symptoms: Moderate neck pain, mild fever, fatigue, palpitations, sweating. Onset 18 days after 2nd dose
**Lab tests at baseline**^a^		
TSH < 0.05 mcU/mL FT4 35 pg/ml FT3 8.26 pg/ml TPOAb, TgAb, TRAb all negative CRP 40 mg/l ESR 91 mm	TSH < 0.05 mcU/ml FT4 25.6 pg/ml FT3 5.5 pg/ml TPOAb, TgAb, TRAb all negative CRP 25 mg/l	TSH 0.027 FT4 35.2 pg/ml TPOAb, TgAb, TRAb all negative CRP elevated ESR elevated Thyroglobulin 338 ng/ml
**Ultrasound**		
Hypoechoic gland, increased volume, inhomogeneous, 35 mm left nodule, reduced vascular flow (Fig. 1A)	Hypoechoic gland, anechoic areas in both lobes, reduced vascular flow	Hypoechoic and inhomogeneous gland, reduced vascular flow
**Scintigraphy**		
Almost absent uptake (Fig. 1C)	n.a.	n.a.
**Therapy**		
Prednisone 25 mg, Propranolol 40 mg; tapered and discontinued after 8 weeks	LT4 discontinuation. Prednisone 25 mg, Propranolol 40 mg, Ibuprofen; tapered and discontinued after 10 weeks	Prednisone 25 mg; tapered and discontinued after 4 weeks
**Lab tests at follow-up**		
after 6–8 weeks		
TSH 10.5 mcU/ml FT4 2.5 pg/ml	TSH 0.07 mcU/ml FT4 16.2 pg/ml FT3 2.97 pg/ml CRP 1.3 mg/l ESR 17 mm	TSH 0.5 FT4 18 pg/ml
after 10–12 weeks		
TSH 6.4 mcU/ml FT4 11 pg/ml	TSH 0.3 mcU/ml FT4 12.5 pg/ml FT3 3.5 pg/ml	
after 14–16 weeks		
TSH 3.48 mcU/ml FT4 9.9 pg/ml	TSH 0.06 mcU/ml FT4 19 pg/ml FT3 4 pg/ml	
**Comment**		
Transient hypothyroidism was not treated and spontaneously resolved. At follow up US echogenicity was still inhomogeneous, but no nodules nor thyroid enlargement were reported (Fig. 1B)	After steroid discontinuation, the patient had a mild symptomatic and biochemical relapse, thus Prednisone was reintroduced with subjective relief	After 4 weeks of therapy, symptoms and thyroid function tests improved, therapy was discontinued, and patient was instructed to undergo periodic screening for thyroid function tests

^a^Normal laboratory values for case 1 and case 2: TSH 0.3–4.2 mcU/ml, FT4 9.3–17 pg/ml, FT3, 2–4.4 pg/ml, CRP < 5 mg/l, ESR < 20 mm. Normal laboratory values for case 3: TSH 0.4–4.4 mcU/ml, FT4 12–22 pg/ml, Thyroglobulin 1,4–78 ng/ml. n.a. = not available

### Search strategy

In this systematic review, we adopted procedures consistent with the Preferred Reporting Items for Systematic Reviews and Meta-Analyses (PRISMA) statement [22]. Data were retrieved from a search of electronic databases including PubMed, Google Scholar, Embase and Scopus; the search was updated to the final article submission date, and it was performed without language restrictions. The search string included combinations of specific terms: (“SARS-CoV-2” OR COVID) AND (Vaccine or Vaccination) AND (“De Quervain thyroiditis” OR “subacute thyroiditis”).

### Study selection

Original articles, Case Report and Case Series recording data on SAT in patients with recent (within 4 weeks) injection of SARS-CoV-2 vaccine shot were eligible for inclusion. Other types of articles (e.g., letter to the editor, congress abstract, comment) were considered eligible for inclusion only if published in international peer-reviewed Journals and if they reported sufficiently documented original cases.

**Fig. 2:**
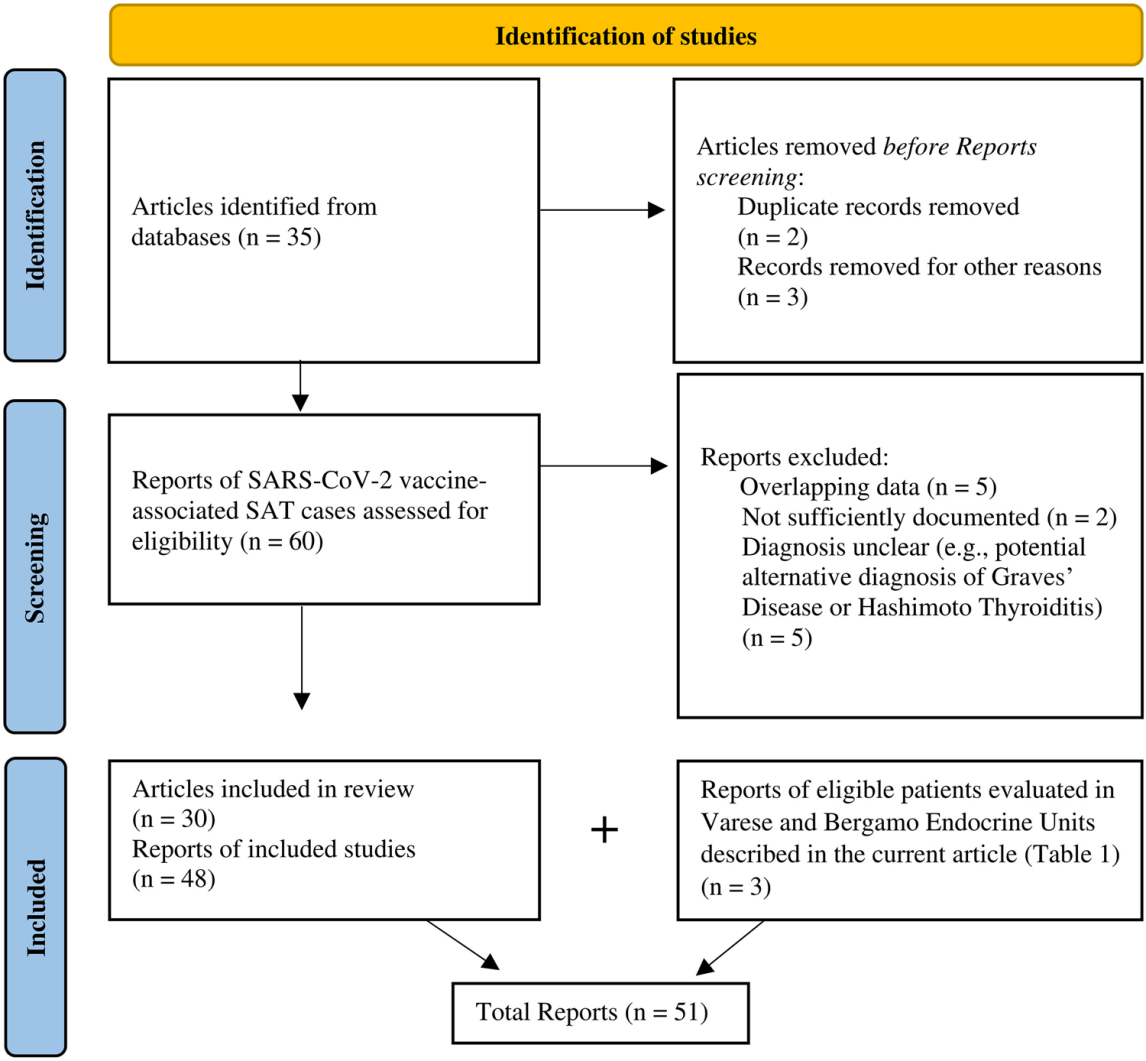
Flow diagram. Flow diagram of recorded studies and reports, according to PRISMA 2020 flow diagram [22] for systematic reviews

Main exclusion criteria were: (1) articles with overlapping patient data; (2) articles reporting SAT cases diagnosed after 4 weeks from vaccine injection; (3) cases in which SAT diagnosis was not sufficiently documented; (4) cases in which diagnoses of SAT was unclear: e.g., if alternative diagnosis of Graves’ Disease or Hashimoto Thyroiditis could not be ruled out. Two Authors (SI, MLT) individually reviewed the retrieved studies, applying the inclusion and exclusion criteria. Disagreements were solved after a final consultation. Figure 2 illustrates the flow diagram of the studies and reports included for analysis.

### Data extraction

For each included article, we recorded reference data (Authors, journal, year of publication, PMID or DOI). For each report, we recorded demographics, history of previous thyroid diseases, type of vaccine, timing of SAT onset, baseline and follow-up laboratory exams, data on diagnostic tools (primarily ultrasound and/or scintigraphy), and therapies performed. Laboratory tests were recorded in terms of thyroid function (hypothyroidism, euthyroidism, thyrotoxicosis) and positive (elevated) or negative (normal) results for thyroxine (T4), triiodothyronine (T3), C-reactive protein (CRP), erythrocyte sedimentation rate (ESR), thyroperoxidase antibodies (TPOAb), thyroglobulin antibodies (TgAb), thyroid receptor antibodies (TRAb); to compare T4, T3, CRP and ESR levels, in order to adjust for different instrument and kit used, we calculated fold change ratio between the measured value and the upper limit of normal (ULN) reported for that specific kit. Patients’ nationality was reported as it was, if available in the article, or derived by Authors’ affiliation Country, if not specified. Data extracted from the reports were almost complete for the baseline outcomes, for the early (4-to-8-weeks) follow up thyroid function tests were reported in 74.5% of patients: for these data we consider the risk of bias to be negligible; however, late (8-to-16-weeks) follow-up data were only reported for 20% of patients, denoting a significant risk of bias due to missing results, thus this bias was carefully considered in evaluating results.

### Statistical analysis

Continuous variables are presented as median and interquartile range (IQR) and were compared using the Wilcoxon rank-sum test to perform pairwise comparisons or the Kruskal–Wallis to compare more than two groups. Categorical variables are presented as numbers and percentages and were compared using the chi-squared test.

Univariate linear regression was used to determine the relationship between continuous variables.

All comparisons were considered statistically significant in case of *p*-value < 0.05. Statistical analyses were performed with STATA version 15 (StataCorp LLC, College Station, Texas).

## Results

### Retrieved articles

According to the above-mentioned search strategy, 35 articles were initially found. Among these, 5 were excluded and a total of 30 articles reporting data on 48 patients were included in the systematic review [17, 23–51]. Study flow-chart is depicted in Fig. 2. With the two additional patients evaluated in Varese and the patient evaluated in Bergamo Endocrine Units (presented in Table 1 and Fig. 1), a total of 51 cases of SAT after SARS-CoV-2 vaccination were retrieved.

### Epidemiological and baseline characteristics of the study population

**Fig. 3:**
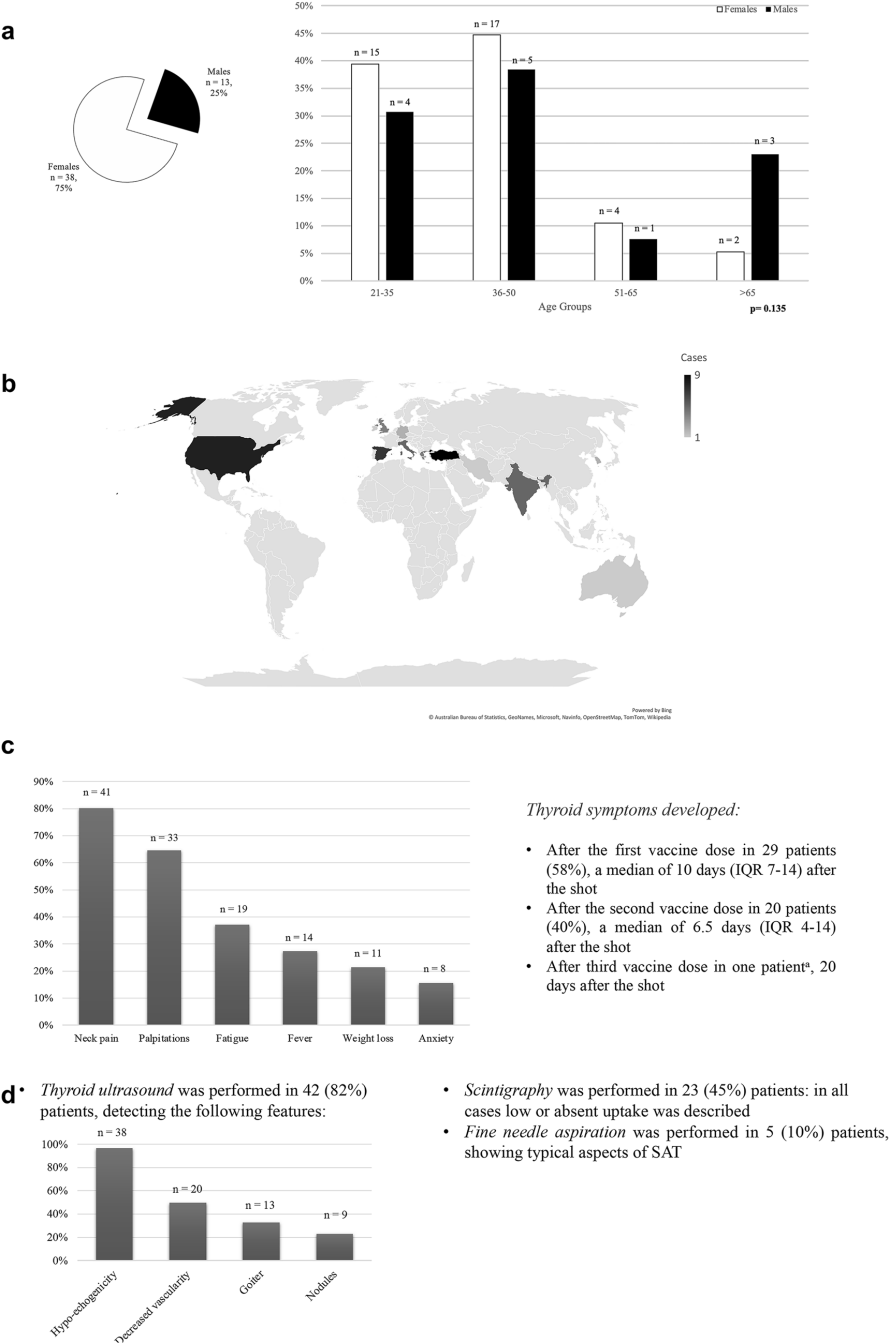
Epidemiology, clinical, and diagnostic approach in SARS-CoV-2 post-vaccination SAT patients. **a** Sex prevalence and age distribution according to sex. Pie chart depicting absolute sex prevalence of reported cases; bar graph showing the relative sex prevalence for female (white bars) and male (black bars), according to four age groups: 21–35 years, 36–50 years, 51–65 years, over 65 years, at the top of each bar the absolute number is reported. p assessed with Wilcoxon rank-sum test. **b** Geographic distribution. Color intensity represents the number of cases reported for that specific Country. **c** Prevalence of SAT symptoms in recorded cases, at the top of each bar the absolute number is reported; bullet point summarizes the timing of symptoms onset according to the vaccine dose. ^a^This patient received 2 doses of inactivated virus vaccine and a subsequent booster of mRNA vaccine. **d** Workup and main diagnostic outcomes of reported patients

Of the 51 patients retrieved, 38 (74.5%) were women, median age was 39.5 years (IQR 34–47), and age did not vary according to sex (p = 0.135) (Fig. 3a). 24 patients (47%) were European, 18 patients (35.3%) Asian, 8 (15.7%) North American and 1 (2%) Australian; Fig. 3b represents geographic distribution. There was no significant difference in age or sex distribution according to different origin groups (all *p* > 0.05). History of thyroid disease was reported in 6 (11.8%) patients: specifically, 5 patients suffered from hypothyroidism under adequate substitution therapy control, of which autoimmune thyroiditis was previously diagnosed in 4 of them, and 1 patient had a history of hemithyroidectomy for a benign thyroid nodule.

Most patients (*n* = 33, 66%) were vaccinated with a mRNA vaccine (Pfizer/BioNTech BNT162b2 or Moderna mRNA-1273), 9 (18%) received viral vector vaccine (Vaxzevria ChAdOx1), 6 (12%) inactivated virus (Sinovac Biotech CoronaVac or Bharat Biotech BBV152), and 2 (4%) underwent heterologous vaccination. Specifically, one patient from Italy received first Vaxzevria vaccine and then Pfizer BioNTech, one patient from Turkey received two doses of Sinovac Biotech and then a booster of Pfizer BioNTech vaccine; in both cases SAT followed mRNA vaccine shot. Patients developed SAT after a median of 10 days (IQR 4–14) after the vaccine shot, and timing of onset after dose did not vary whether it was the first or second dose (*p* = 0.287). Main symptoms reported were neck pain, palpitations, fatigue, fever, weight loss, anxiety, or insomnia (Fig. 3c); 2 patients (4%) were asymptomatic, discovering thyroid dysfunction at routine laboratory exams.

#### Management, therapy, and outcomes

Baseline thyroid exams revealed thyrotoxicosis, primarily defined by TSH suppression, in 45 (88.2%) patients; in 84.4% of them, T4 was also elevated as compared to the ULN, with a median fold change of 1.6 (IQR1.3–2.1); similarly, T3 was elevated in 67.9% of patients, and median fold change over ULN was 1.6 (IQR1.3–2.3). Excluding the 4 patients with known autoimmune thyroiditis, TPOAb were positive in 8.7% of patients and TgAb in 15.7%, whereas no one developed TRAb. CRP was elevated in 28 of 33 patients (84.8%) with a median fold change over ULN of 8 (IQR 4.6–18.2); ESR was elevated in 29 of 33 patients (87.9%), tripled if compared to ULN, with a median fold change of 3.05 (IQR 2.1–4.4). At univariate linear regression, higher ESR values correlated with thyrotoxicosis severity (significantly for T4, *p* = 0.035; with a trend towards significance for T3, *p* = 0.07), whereas CRP did not correlate with hyperthyroidism severity.

In terms of diagnostic tools employed (Fig. 3d), 42 (82.3%) patients underwent ultrasound, Fig. 3d depicts the main ultrasound features reported, and Fig. 1a-b depicts an emblematic case of ultrasound performed in a patient with SARS-CoV-2 vaccine-associated SAT evaluated in Varese. Scintigraphy was performed to confirm diagnosis in 23 (45.1%) patients, in all cases low or absent uptake confirmed the destructive nature of thyroiditis, Fig. 1c depicts an emblematic case of scintigraphy performed in a patient with SARS-CoV-2 vaccine-associated SAT evaluated in Varese. Fine needle aspiration (FNA) was performed in 5 (9.8%) patients and reported typical aspects of SAT: epithelioid granulomas, multinucleated giant cells, aggregate of histiocytes, lymphocytes and neutrophils.

Data on therapy management were available for 49 patients, 10 (20.4%) did not receive any medication for SAT; patients were treated with nonsteroidal anti-inflammatory (NSAIDs) in 66.7%, beta-blockers in 56.4%, corticosteroids (prednisone, prednisolone or methylprednisolone) in 56.4%, and one patient received methimazole. Patients previously taking levothyroxine for hypothyroidism, discontinued therapy temporarily. Therapy was continued for a median of 4 (IQR 3.8–6) weeks.

At a 4-to-8-week follow-up, thyroid function assessment was available for 38 patients: of them, 16 (42.1%) were euthyroid, 10 (26.3%) hypothyroid, and 12 (31.6%) were thyrotoxic. Among the hypothyroid patients, 6 (60%) received LT4 therapy. Approximately half of the patients with suppressed TSH had elevated T4 (45.5%) and T3 (50%), median fold change over ULN was 1.7 (IQR 1.2–1.8) for T4 and 1.2 (IQR 1.1–1.3) for T3. CRP was reassessed in 15 patients, 4 of them (26.7%) still had high values, with a fold change median over ULN of 3 (IQR 1.9–3.9); ESR at follow-up was available for 14 patients, 4 of them (28.6%) had high ESR, with a fold change median over ULN of 1.8 (IQR 1.4–2.2).

At a 8-to-16-week follow-up, thyroid function exams were available for 10 patients: 4 (40%) were euthyroid, 4 (40%) hypothyroid, while 2 patients (20.0%) presented TSH suppression: one was a case of improving but persistent mild thyrotoxicosis [51] and the other was a case of thyrotoxicosis relapse after discontinuation of steroid therapy (Table 1, Case 2).

### Differences in epidemiological and baseline characteristics according to vaccine type

We compared epidemiological and baseline characteristics of patients according to the type of vaccine received: mRNA, viral vector, or inactivated virus (Table 2**)**. We then identified if there were differences in mRNA-based *vs.* viral vector or inactivated virus vaccines. No significant differences were observed in terms of sex proportion and age distribution (*p* = 0.248 and *p* = 0.473, respectively); there was a significant difference in geographic origin (*p* = 0.019), mostly because inactivated virus vaccines are not approved by the Food and Drug Administration (FDA) and the European Medicine Agency (EMA), thus they are mostly used in eastern countries, where approved. No differences were found according to timing of onset after the shot (*p* = 0.549). Symptoms were similar among vaccines (all *p* > 0.05), except for weight loss, which was reported in only 4 cases (11.4%) in the mRNA vaccine group and in 6 cases (40%) in the non-mRNA vaccine group (*p* = 0.021). Baseline thyroid function and inflammation indexes did not vary according to vaccine type (all *p* > 0.05). All 6 cases of history of previous thyroid disease belonged to the mRNA vaccine group.

**Table 2:** Comparison of demographic and clinical features according to different SARS-CoV-2 vaccine categories

No., %	mRNA vaccines 35, 70%	Non-mRNA vaccines			p-value*
		Viral vector vaccines^a^ 9, 18%	Inactivated virus 6, 12%		
Sex F (No., %)	25, 71.4%	8, 88.9%	5, 83.3%		0.248
Age (median, IQR)	40.0, 34–44	47, 39–55	36, 34–38		0.473
European (No., %)	19, 54.3%	5, 55.6%	0		**0.019**
North American (No., %)	7, 20%	0	0		
Asian (No., %)	8, 22.9%	4, 44.4%	6, 100%		
Australian (No., %)	1, 2.9%	0	0		
After 1st dose (No., %)	20, 57%	8, 88.9%	2, 33.3%		0.701
After 2nd dose (No., %)	14, 40%	1, 11.1%	4, 66.7%		
After 3rd dose (No., %)	1, 3%	0	0		
Onset (days after vaccine shot—median, IQR)	9, 4–14	14, 10–14	7, 4–14		0.549
Neck pain (No., %)	26, 74.3%	8, 88.9%	6, 100%		0.123
Palpitations (No., %)	24, 68.6%	4, 44.4%	4, 66.7%		0.304
Fatigue (No., %)	13, 37.1%	2, 22.2%	4, 66.7%		0.849
Fever (No., %)	9, 25.7%	2, 22.2%	2, 33.3%		0.944
Weight loss (No., %)	4, 11.4%	2, 22.2%	4, 66.7%		**0.021**
Anxiety (No., %)	6, 17.1%	2, 22.2%	0		0.736
Baseline TSH suppression (No., %)	32, 91.3%	8, 88.9%	4, 66.7%		0.254
Baseline T4 increase					
(No., %)	26, 74.3%	8, 88.9%	3, 50%		0.944
(median fold change over ULN, IQR)	1.6, 1.4–1.9	1.3, 1.2–3.3	1.8, 1.7–2.7		0.971
Baseline T3 increase					
(No., %)	12, 54.5%	3, 60%	4, 66.7%		0.618
(median fold change over ULN, IQR)	1.5, 1.3–2.1	1.4, 1.1–2.7	1.9, 1.6–2.6		0.499
TPOAb	7, 21.2%	1, 12.5%	0		0.276
TgAb	8, 28.6%	2, 40%	0		0.597
Baseline CRP increase					
(No., %)	19, 90.5%	5, 83.3%	4, 66.7%		0.233
(median fold change over ULN, IQR)	8.4, 4.6–18.4	5.8, 5.7–6.9	10.9, 6.0–15.5		0.699
Baseline ESR increase					
(No., %)	19, 90.5%	5, 83.3%%	5, 83.3%		0.545
(median fold change over ULN, IQR)	3.1, 2.2–4.4	2.8, 1.9–3.2	3.9, 2.7–6.7		0.885

*p-value assessed by Wilcoxon rank-sum test (for continuous variables) or chi-squared test (for categorical variables), comparing mRNA *vs.* non-mRNA vaccines. ^a^Every patient in this category received Vaxzevria ChAdOx1, no cases of SAT following Janssen Ad26.COV2.S vaccine were reported

## Discussion

In the last year, after the beginning and worldwide massive diffusion of SARS-CoV-2 vaccination, there has been an increasing number of reports suggesting an association between thyroid dysfunctions and SARS-CoV-2 vaccines administration.

We hereby performed a systematic review reporting and analyzing data from 51 patients (48 published reports, 3 original cases evaluated by Authors) developing SAT after SARS-CoV-2 vaccination, to shed a light and add concrete evidence about this possible relationship.

Our first objective was to evaluate the demographical and clinical characteristics at presentation in this setting of patients. Considering this first aim, epidemiological and clinical features of patients affected by post-vaccination SAT seem similar to the classic form of virus-related SAT, as compared to a large cohort study [52], with a strong female prevalence, and a median age of onset at 40 years, with most prominent symptoms being neck pain, palpitations, fatigue, fever, weight loss. As for laboratory analyses, reported patients were usually thyrotoxic, and presented raised serum inflammation markers, particularly ESR, which correlated with thyrotoxicosis severity. Ultrasound, scintigraphy or FNA confirmed, when performed, typical features of classic SAT. Treatment strategies encompassed NSAIDs, beta-blockers and steroids, in line with American Thyroid Association guidelines [53]. We treated our three patients with prednisone, besides symptomatic therapy, due to the major severity of symptoms and/or the severity of thyrotoxicosis. This decision was pondered after raising ourselves the question if steroid therapies could impair vaccine-induced immune response. The three original patients hereby described developed thyroiditis after at least 2 weeks from vaccine shot, therefore we concluded that it was safe to employ steroids since the vaccine-induced immune response should have already been established. Further studies are needed to evaluate if an oral therapy with steroid, at what dosage and timing, may affect vaccine efficacy. As a precaution, until this subject is clarified, it could be suggested that if SAT onset occur in the first days after vaccine shot, it would be cautious to avoid or delay, if possible according to symptoms and thyrotoxicosis severity, steroid treatment.

Our second objective was to evaluate whether mRNA vaccines, only recently approved for emergency use authorization, have a peculiar pattern in determining this side effect as compared to viral vector or inactivated virus vaccines. Noticeably, mRNA-based vaccine uses lipid nanoparticles to facilitate mRNA transport into cells and contain several excipients and lipids, including polyethylene glycol (PEG), which might induce immune response in predisposed individuals [54] and has been linked with cases of anaphylactic reaction to mRNA vaccine [55]. Peculiarly, all patients with a previous diagnosis of autoimmune thyroid disease who developed SAT belonged to the mRNA vaccine group, possibly identifying a predisposition to develop SAT after mRNA-based SARS-CoV-2 vaccine secondary to ASIA syndrome, which is often associated to a personal or familial history of autoimmune diseases [19], but further studies and larger cohorts are needed to verify this suggestion.

In the face of several billion vaccine doses that have been administered globally so far [56], 51 recorded SAT cases following SARS-CoV-2 vaccination seem to define a very uncommon side effect. It is possible, however, that incidence of SAT could be underestimated, and that the paucity of cases described is not due to the rarity of this association but rather to a lack of awareness of this condition. SAT symptoms, despite peculiar, may indeed be confounded for post-vaccination general systemic side effects and could resolve spontaneously with symptomatic therapy, before a specialist may define diagnosis. At the same time, it is not entirely feasible to rule out other possible SAT etiologies (e.g., viral infections), however the reported cases all had a strong temporal association with vaccination schedule and cases did not clear peaked in the summer season, as expected for viral SAT (data not shown) [1]; moreover, some authors performed other tests to rule out other etiologies. Pujol et al [42], in an exemplary way, first ruled out SARS-CoV-2 associated SAT by detecting negative IgG anti-N, which are specific for natural (and not vaccine-driven) immunity, then performed molecular search of the principal pathogens associated with SAT in the FNA sample, which resulted negative.

We believe that SARS-CoV-2 vaccine-associated SAT deserves to be acknowledged in order to (I) avoid inappropriate treatment: physicians should treat symptomatic patients with NSAIDs, beta-blockers in case of palpitations, and/or steroid in case of unresponsiveness or if symptoms are severe; in the latter case it could be suggested to introduce steroids after two weeks post vaccination, if possible; (II) monitor thyroid morphology with ultrasound, which is an inexpensive and harmless diagnostic tool that could confirm diagnostic suspicion; (III) identify the hypothyroidism phase, which usually follows the destructive thyrotoxic phase: since it is usually a self-limiting condition, it should be monitored and treated only in case of severe symptoms or severe hypothyroidism; (IV) schedule a middle/long term follow-up for thyroid function since in about 5–15% of patients, hypothyroidism can be permanent [57, 58], and a life-long thyroid replacement therapy be required; (V) notify similar cases as possible adverse events of vaccination for pharmacovigilance purposes.

In conclusion, SARS-CoV-2 vaccine-associated SAT is a novel entity, which shares epidemiological and clinical characteristics with the classic post-viral SAT but could be triggered by peculiar mechanisms. It is possible that patients with a previous history of thyroid disease may be more prone to develop SAT after mRNA vaccines, but further studies and larger cohorts are needed to verify this suggestion. Most importantly, since SARS-CoV-2 vaccine-associated SAT it is usually of mild/moderate severity and could be treated in most cases with symptomatic therapy or a short course of steroids, it should not raise any concern regarding the need to be vaccinated, since the risks of COVID-19 undoubtedly outweigh the risks of the vaccination.

We believe that our work would increase awareness on this condition, to identify and properly treat patients that should be also monitored for possible long-term hypothyroidism.
